# Transcriptomic insights into the immune responses of the lung and muscle of non-healthy harbor porpoises (*Phocoena phocoena*)

**DOI:** 10.3389/fimmu.2026.1738836

**Published:** 2026-03-09

**Authors:** Eda Merve Dönmez, Andrej Fabrizius, Ursula Siebert

**Affiliations:** 1 Institute of Animal Cell and Systems Biology (IZS), University of Hamburg, Hamburg, Germany; 2 Institute for Terrestrial and Aquatic Wildlife Research (ITAW), University of Veterinary Medicine Hannover, Foundation, Büsum, Germany

**Keywords:** *de novo* assembly, harbor porpoise Phocoena phocoena, immune system – respiratory tract, lung, marine mammals, muscle, pathophysiology, transcriptome

## Abstract

**Introduction:**

The harbor porpoise populations of the North and Baltic Seas are highly impacted by human activities, including underwater-radiated noise, fisheries and pollution. These cumulative stressors can have various detrimental effects, such as reduced foraging success, altered behavior and an impaired immune system. Harbor porpoises especially suffer from diseases of the respiratory tract which are partly caused or exacerbated by high parasitic prevalence in the lungs that may ultimately affect diving ability and competitiveness due to insufficient oxygen uptake and supply to the locomotor musculature.

**Methods:**

To investigate pathophysiological mechanisms and potential compensatory adaptations to pathogenic insults, we employed transcriptomics and compared lungs and muscles of harbor porpoises with compromised respiratory health to healthy individuals. Additionally, a *de novo* transcriptome assembly was generated to identify transcripts that may be involved in immune-related responses.

**Results and Discussion:**

Non-healthy harbor porpoises showed a distinct host-pathogen defense reaction in the lung, suggesting similarities to immune responses of humans suffering from lung diseases, which may be conserved along the mammalian lineage despite vastly different habitats. However, the lung transcriptomes did not indicate a Th2 immune response which is typically activated upon parasitic insults. Severely infected harbor porpoises may be overwhelmed or weakened by prolonged parasitic exposure and immune activation, possibly affecting simultaneous pathogenic clearance and tissue repair. The muscles of non-healthy harbor porpoises exhibited enhanced stress signaling and tightly regulated tissue degradation/regeneration, potentially reflecting a chronic inflammation state. Higher expression of hypoxia- and oxidative stress-associated transcripts in the muscle were consistent with hypoxia-induced transcriptional patterns and suggest a systemic pathological challenge. The *de novo* assembly identified significantly dysregulated non-coding RNAs in the lung and muscle which may be associated with regulatory processes. Several transcripts of the *de novo* assembly remained unidentified, thus their putative function needs to be elucidated. In marine mammals, the understanding of molecular immune responses still remains incomplete. This is the first study to describe the lung transcriptome of wild harbor porpoises in regard to pathophysiology. These insights contribute to the understanding of the interaction between anthropogenic impacts, infectious diseases and molecular immune responses in cetaceans, thus supporting cetacean health assessments and conservation efforts.

## 1 Introduction

The harbor porpoise (*Phocoena phocoena*) is one of the most common and widely distributed cetacean species in European seas, and the only one native to German waters. While not of concern on a global stage, subpopulations, such as the Baltic Sea Proper harbor porpoises, are indexed on the IUCN Red List of Threatened Species (1, 2). Recent studies have also noted a worrying decline in porpoise numbers for the German part of the North Sea (3). Due to their preference for coastal habitats, harbor porpoises are constantly exposed to natural and human-made environmental changes (4). The North and Baltic Seas are notoriously frequented by maritime traffic and experience a surge in recreational and industrial interest (e.g., offshore construction, military and seismic surveys; (5–9)). Chemical and industrial flow-off can wash contaminants and toxic metals into the sea which biomagnify in apex predators such as the harbor porpoise (10–12). Bioaccumulation of these reagents has been suspected to impair the reproduction of harbor porpoises (13–15). Additionally, neonates are impacted to some degree, as females offload a considerable amount of accumulated contaminants to their calves (16). The continuous exposure to contaminants also impairs the immune system by acting as immunosuppressant and hence, promotes pathogen contraction and inhibits immune defense responses (12, 17, 18). In the last decades, harbor porpoises from German waters have shown declining health states compared to Arctic and Scandinavian populations that live in less humanly frequented areas (18–21). While harbor porpoises may be able to cope with mild levels of parasitic infestation, they show increasingly broader parasitic diversity and load (22, 23). Especially the respiratory tract seems to be targeted which exhibits accumulating pathological and inflammatory lesions that are associated with lungworm prevalence and contraction of secondary bacterial infections (18, 20, 24–27). Studies have hypothesized that such parasitic infestations and pathological lesions may hinder lung function due to tissue damage and parasitic obstruction of air and blood flow (20, 21, 28, 29).

With harbor porpoises only utilizing approximately 60% of their complete lung capacity (30, personal communication with U. Siebert), further impairment of the lung tissue function may severely aggravate their ability to compensate these detrimental impacts. Although harbor porpoises tend to preferably dive shallow and short (31), telemetry studies have observed dives up to 200–400 m, if the bathymetry allows for such depths (32, 33). These deeper dives are usually performed to forage (31, 34), or spontaneously to avoid excessive underwater-radiated noise (35, 36). Deterrence by exceeding noise levels can also cause a prolonged stay at deeper sea levels (34, 35), which may put stressed harbor porpoises at higher risk of gas embolism (reviewed in 37), if oxygen levels are insufficient to support unplanned, breath-hold dives (38).

Studies in humans have confirmed negative effects on the muscle stemming from diseases of the lung, such as bronchopneumonia, chronic obstructive pulmonary disease (COPD) and pulmonary fibrosis (28, 39, 40). While major immune responses often are conserved among the mammalian lineage, marine mammals may possess differing mechanisms to adapt to their life in the aquatic environment (41, 42).

In order to understand the described mechanism better, we performed a comparative transcriptome analysis of the lung and main locomotor muscle of wild harbor porpoises from the North and Baltic Seas against the recently published harbor porpoise reference genome (Scottish population; 43). We compared harbor porpoises with impaired and good respiratory health to further understand molecular compensatory mechanisms for reduced oxygen uptake caused by respiratory lesions. Additionally, we generated a multi-tissue *de novo* transcriptome assembly (brain, lung, liver and muscle) to detect novel transcripts that were potentially involved adaptive immune and stress responses.

## 2 Materials and methods

### 2.1 Animals and sampling

The harbor porpoises used in this study stranded dead or died after live-stranding or as bycatch between 2016 and 2022 in Schleswig-Holstein, Germany. Tissue samples were collected during routinely conducted necropsies at the Institute of Terrestrial and Aquatic Wildlife Research (ITAW), University of Veterinary Medicine Hannover, Foundation, Büsum, Germany, which is part of the German stranding network (44, 45). Full necropsies and further investigations were conducted on all individuals according to standardized protocols (20, 46). Based on the summary of findings, animals were categorized into healthy (n = 5) and non-healthy (n = 13) individuals with regard to their pulmonary health status. Non-healthy animals displayed pathological lesions due to moderate or severe lungworm infestations and bacterial infections and suffered or died from bronchopneumonia (**Table 1**). Healthy individuals were in overall good pulmonary health (**Table 1**).Tissue samples of the skeletal muscle (*Musculus longissimus dorsalis*), lung, liver and brain (cerebellum and visual cortex) were either subsampled from archived samples or freshly sampled and immediately preserved in RNA stabilization solution (NucleoProtect RNA, Macherey-Nagel, Düren, Germany). All samples were then stored at -80 °C until subsequent usage.

**Table 1:** Metadata of the wild harbor porpoises used in this study.

Individual	Experiments	Condition	Sex	Sampling year	Location	Age class	Bycatch	Nutrition	Disease
Pph_h1	qRT-PCR	healthy	male	2020	BS	neonate	no	nd	perinatal death
Pph_h2	RNA-Seq (muscle)	healthy	male	2020	BS	neonate	suspected	nd	none
Pph_h3	RNA-Seq (lung, muscle), qRT-PCR	healthy	male	2021	BS	juvenile	yes	G	mild inflammation in the lung, stomach and liver
Pph_h4	DNA, RNA-Seq (lung, muscle, brain), qRT-PCR	healthy	female	2022	NS	neonate	no	G	perinatal death
Pph_h5	qRT-PCR	healthy	female	2022	BS	adult	no	G	suspected septicemia (*P. multocida*)
Pph_nh1	RNA-Seq (muscle)	non-healthy	female	2019	BS	adult	suspected	M	bronchopneumonia, hepatitis, adrenalitis, final septicemia (*P. multocida*)
Pph_nh2	RNA-Seq (muscle), qRT-PCR	non-healthy	male	2019	NS	juvenile	no	M	bronchopneumonia, gastroenteritis
Pph_nh3	DNA, RNA-Seq (lung, liver), qRT-PCR	non-healthy	male	2022	BS	juvenile	yes	G	bronchopneumonia, gastritis
Pph_nh4	DNA, RNA-Seq (lung, muscle), qRT-PCR	non-healthy	female	2022	NS	adult	no	P	meningoencephalitis, bronchitis, gastritis
Pph_nh5	RNA-Seq (muscle)	non-healthy	male	2022	BS	juvenile	yes	G	bronchopneumonia, gastritis, endoparasitosis
Pph_nh6	RNA-Seq (muscle)	non-healthy	male	2021	BS	juvenile	yes	G	bronchopneumonia, gastritis
Pph_nh7	RNA-Seq (muscle)	non-healthy	male	2020	NS	juvenile	no	P	bronchopneumonia, dermatitis
Pph_nh8	DNA, RNA-Seq (muscle), qRT-PCR	non-healthy	male	2019	NS	adult	no	P	bronchopneumonia, gastritis
Pph_nh9	qRT-PCR	non-healthy	female	2016	BS	adult	no	P	septicemia (red murrain)
Pph_nh10	qRT-PCR	non-healthy	female	2016	BS	adult	yes	P	bronchopneumonia
Pph_nh11	qRT-PCR	non-healthy	male	2016	NS	juvenile	no	P	cachexia, bronchopneumonia
Pph_nh12	qRT-PCR	non-healthy	female	2021	BS	adult	yes	G	bronchopneumonia, endoparasitosis
Pph_nh13*	qRT-PCR	non-healthy	female	2022	BS		suspected		bronchopneumonia, hepatitis, nephritis, dermatitis

* Condition, sex, cause of illness and death was unknown during performance of experiments. Shown are the year of the necropsy (sampling year) and location, health condition after assessment including diagnosed diseases, sex, age class, nutrition state and bycatch. Further data includes the experiments for which the samples were used. DNA, *De Novo* Assembly; RNA-Seq, RNA-Sequencing; qRT-PCR, quantitative real-time PCR; BS, Baltic Sea; NS, North Sea; nd, not determined; P, poor; M, moderate; G, good.

### 2.2 RNA isolation and quality control

Total RNA was extracted with the RNeasy Mini Kit (Qiagen, Hilden, Germany) in accordance with the manufacturer’s instructions. The initial homogenization step with the kit’s RLT buffer was replaced with a phenol-chloroform extraction to maximize yield and purity of the isolated RNA. Tissue samples (20–30 mg) were minced and homogenized by bead beating in 1 mL of Trifast reagent (PEQLAB, Erlangen, Germany). RNA was isolated from the homogenates by phase extraction using chloroform and ethanol (70%). Thereafter, the kit manual was followed and an additional 15 minute on-column DNA I digest (Qiagen, Hilden, Germany) step was conducted as recommended. RNA concentration and quality, indicated by the RNA Integrity Number (RIN), was assessed using the Agilent TapeStation System (Agilent Technology, Santa Clara, CA, United States). RIN scores of the samples varied from 4.8 to 7.0.

### 2.3 Sequencing and quality trimming

The RNA-Seq library preparation (NEBNext Ultra II Directional RNA library prep kit for Illumina, New England Biolabs, Ipswich, MA, USA) for paired-end sequencing of 2 x 150 nt was generated from 5000 ng RNA. Sequencing was performed on an Illumina NextSeq 2000 platform (StarSEQ, Mainz, Germany) with an output of 25 million reads per sample. Initial sequence quality control and trimming were carried out on the Galaxy server (version 21.09) of the University of Hamburg Biology department. Quality control was assessed using the FastQC v 0.73 and MultiQC v 1.11 tool on Galaxy. The first 20 5’-terminal nucleotides and Illumina adapter sequences (mismatch count = 2, internal match = 10) were cropped from the raw reads with Trimmomatic v 0.38.1. Reads below the length of 20 nucleotides were discarded and a minimum average quality value of 20 was required for consideration.

The raw sequence files are available at the NCBI Sequence Read Archive (SRA) from (SRA BioProject ID: PRJNA977857).

### 2.4 Differential expression analysis via RNA-Seq

The trimmed sequences of the lung and muscle transcriptomes were mapped and aligned against the harbor porpoise reference genome (mPhoPho1.1, released June 2024, RefSeq Accession: GCF_963924675.1) using HISAT v 2.2.1 and featurecounts v 1.6.4 (for detailed metrics, see **Supplementary Table 1**). Individual transcripts per million (TPM) for each transcript were calculated with the Galaxy tool “Generate CPM, TPM, RPK” v 0.4.0 and mean values for the non-healthy and healthy animals were calculated. Differentially expressed transcripts (DETs) were determined using DESeq2 v 2.11.40.7 (47).

### 2.5 Validation of the RNA-Seq via quantitative real time PCR

Due to the low sample size, results of the lung transcriptome analysis were validated via qRT-PCR for a panel of transcripts in a larger sample subset (healthy, n = 4 and non-healthy, n = 9; **Table 1**). Of these transcripts, three were significantly upregulated (JCHAIN, GPNMB, OLR1) and one downregulated (QPCT) in non-healthy harbor porpoises in the transcriptome analysis. Based on the transcriptome analysis, the ribosomal protein RPS8 was selected as reference transcript with no regulation for normalization. cDNA was synthesized from 1,000 ng of total RNA, using the RevertAid H--First Strand cDNA Synthesis Kit (Thermo Fisher, USA) and diluted with Ribonuclease-free water (1:1). qRT-PCR was performed on the ABI 7500 real-time PCR system with the Power SYBR Green master mix (Applied Biosystems, Germany) and the following species-specific primer sequences: JCHAINfor-5’- GATGAAGATGAAAGGACTGTTC-3’, JCHAINrev-5’- TCAGAGGAACAATAATTCTGATGT-3’; OLR1for-5’- GATAATCCAATTATCCCAGGTG-3’, OLR1rev-5’- TCTGGTGATGAAGTTCCATTAG-3’; GPNMBfor-5’- CATGATGTGCTGAGCAATGAG-3’, GPNMBrev-5’- GTGCATCACTGGTCAGAAGT-3’; QPCTfor-5’- TCGTTGAAGAATATTTCAGACTCG-3’, QPCTrev-5’- GATGTGCAGTTGATGCCATCT-3’; RPS8for-5’- GCAAGACAAGGATCATTGATG-3’, RPS8rev-5’- CTCAGGAGTCAGCTTGGC-3’. The qRT-PCR settings were adjusted to 95 °C for 15 sec, 58 °C for 60 sec, 72 °C for 30 sec and repeated for 40 cycles. Each sample was applied with three technical replications and relative fold change was calculated based on the ΔΔCT method (48). Statistical analysis was performed in GraphPad Prism v 10. Mean ΔΔCT values were tested for normal distribution, and the Mann-Whitney-U test was conducted for *post-hoc* analysis and statistical significance testing.

### 2.6 Gene ontology analysis

For Gene Ontology (GO) and pathway analysis, only differentially expressed transcripts (DET) with an FDR-corrected p-value (pval_FDR_) ≤ 0.05, a logarithmic fold change (FC_log2_) ≥ 1 or ≤ -1 and a mean TPM_non-healthy_ ≥ 5 were considered for the analysis. Significantly dysregulated transcripts were analyzed for overrepresentation with the PANTHER classification database (v 19.0, reference: human *Homo sapiens*). GO Slim “Biological Processes” were analyzed and child terms with the highest specification were regarded. Results were visualized with the R package of “ggplot2”. Kyoto Encyclopedia of Genes and Genomes (KEGG, v 114.0) pathway analysis was conducted to visualize the differential regulation of transcripts in selected pathways.

### 2.7 *De novo* transcriptome assembly and annotation

Trimmed transcriptomes of the lung, muscle, liver and brain with the best RIN score (n = 1 per tissue, **Table 1**) were chosen to generate the *de novo* transcriptome assembly, for which Trinity v 2.15.2 with the associated tools was used on the High-Performance Computing Linux-Cluster of the University of Hamburg. Pre-assembly quality was checked with FastQC v 0.73 and MultiQC v 1.11 and post-assembly quality was analyzed with BUSCO v 5.6.1 against the databases of Mammalia and Cetartiodactyla. The annotation of the *de novo* assembly was generated with Trinotate v 4.0.2. Blast v 2.16.0 and Diamond v 2.1.11 were used to identify nucleotide- and protein sequence hits against multiple databases (Human EMBL Protein database, UniProt Protein database, UniProt Protein database for the bottlenose dolphin *Tursiops truncatus*, beluga *Delphinapterus leucas*, vaquita *Phocoena sinus*, sperm whale *Physeter macrocephalus*, narwhal *Monodon monocerus*, Indo-Pacific humpback dolphin *Sousa chinensis* and Yangtze finless porpoise *Neophocaena asiaeorientalis*). Also, a cetacean-specific database was generated, for which all cetacean entries of the UniProt Proteome database were included. The transcriptomes of the lungs and muscles were back-mapped with BowTie2 v 2.3.0 against the *de novo* assembly. BowTie2 included RSEM as estimation method and was set to trinity mode with a paired-end reverse strand library. Trinity transcripts were analyzed for differential expression with the R package of DeSeq2 v 1.48.1 and then filtered with the following cut-offs: pval_FDR_ ≤ 0.05, FC_log2_ ≥ 1 or ≤ -1 and mean TPM_non-healthy_ ≥ 5.

Significantly dysregulated Trinity transcript sequences with no annotation via the Trinotate pipeline were extracted and run against the core nucleotide database (core_nt) of NCBI nucleotide blast (Blastn, BLAST+ version 2.17.0) with the organism set to mammalia (taxid ID: 40674) and megablast (highly similar sequences) for optimization. Blast hits were considered reliable, if the query covered ≥ 50% and the sequences shared ≥ 70% identity.

## 3 Results

### 3.1 Enhanced immune defense and reduced ECM processes in the lung

**Figure 1:**
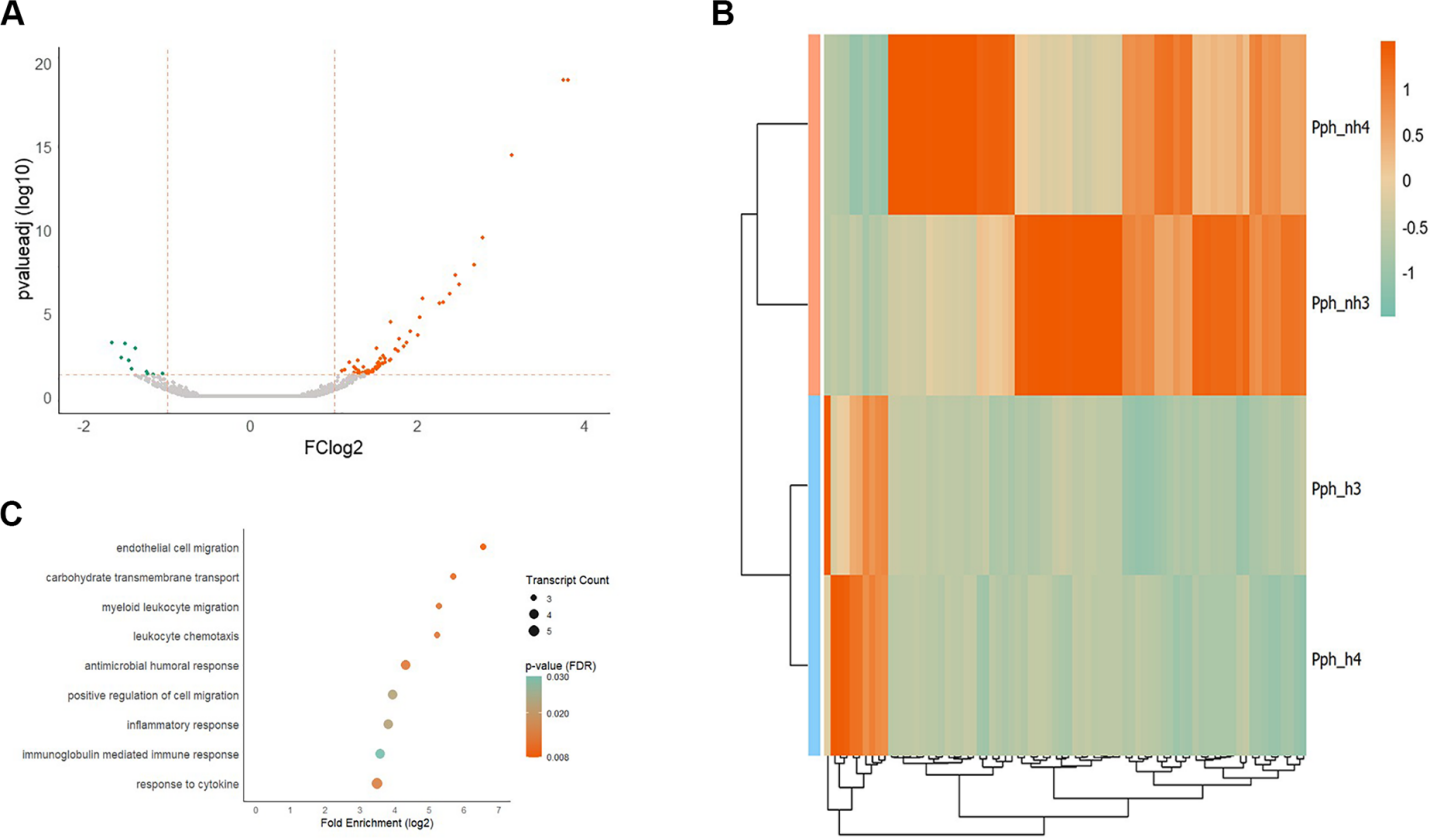
Differential analysis of the lung. **(A)** depicts the significantly dysregulated transcripts in the lung of non-healthy compared to healthy harbor porpoises. Upregulated transcripts are orange, downregulated transcripts are green. The x-axis represents the logarithmic fold change (FC_log2_) and the y-axis indicates the FDR-corrected p-value. For better visualization, the y-axis has been log10-transformed. Dashed orange lines highlight the applied cut-offs for fold change (FC_log2_ ≥ 1/≤ -1) and for FDR-corrected p-value (pval_FDR_ ≤ 0.05). Transcripts were considered significantly dysregulated when pval_FDR_ ≤ 0.05, FC_log2_ ≥ 1/≤ -1 and mean TPM_non-healthy_ ≥ 5. **(B)** Hierarchical clustering of the significant DETs in non-healthy (apricot) and healthy (light blue) harbor porpoises. Upregulated transcripts are highlighted in orange and downregulated transcripts in green (see legend for FC_log2_ values). **(C)** Significant DETs were analyzed for overrepresented GO-Slim terms in the category “Biological Process” with PANTHER (reference: human). Significant enrichment could only be found for upregulated transcripts and are depicted here. The fold enrichment is log2-transformed and the transcript number of the identified terms are indicated by size of the dot. The color of the dots illustrates the significance level from high (orange) to low (green).

With the harbor porpoise genome as reference, over 17,591 transcripts were expressed in at least one individual. After the cut-offs were applied (pval_FDR_ ≤ 0.05, FC_log2_ ≥ 1 or ≤ -1, mean TPM_non-healthy_ ≥ 5), 66 upregulated and 10 downregulated DETs in the lungs of non-healthy harbor porpoises were considered for differential and pathway analyses (**Figure 1A**). Within the significantly dysregulated transcripts, the two non-healthy harbor porpoises exhibited varying expression profiles for a number of DETs (**Figure 1B**). In individual Pph_nh3, 17 DETs were higher expressed compared to individual Pph_nh4. These DETs were mostly involved in immune reactions, specifically immunoglobulin-mediated responses (**Figure 1B**, **Supplementary Table 2**). A total of 19 DETs showed higher elevation in individual Pph_nh4 and encompassed transcripts with a role in stress responses, metabolic processes and surfactant production (**Figure 1B**, **Supplementary Table 2**).

Differentially expressed transcripts in the lungs of non-healthy harbor porpoises mostly showed significant elevation in immune system responses compared to healthy harbor porpoises (**Figure 1C**). This included leukocyte recruitment, immunoglobulin activation and responses to cytokine and inflammation (e.g., IGHG1, JCHAIN, CXCL8, IGKC, CXCL17, PIGR, **Table 2**; **Figure 1C**). Moreover, carbohydrate metabolism was enhanced in non-healthy harbor porpoises (**Figure 1C**, **Table 2**; AQP3, AQP7, CA4, SLC2A3; **Supplementary Table 2**). Several DETs were involved in metal ion binding, especially of iron (HP, IDO1, LTF, MOXD1, S100A12, S100A8, S100A9, STC2; **Supplementary Table 2**). KEGG pathway analyses of dysregulated transcripts in the lungs of non-healthy harbor porpoises showed high upregulation of transcripts involved in the proinflammation of lipid and atherosclerosis, a cardiovascular disease (OLR1, NCF1, CXCL8, SELE, SELP, IL1B, CCL5, MMP3, MMP9; hsa05417, **Supplementary Figure 1**). Also, among the most significantly upregulated transcripts, multiple S100 transcripts (S100A4, S100A8, S100A9, S100A12), surfactant transcripts (SFTPA, SFTPB) and extracellular matrix-associated transcripts (MMP9, TNC, CCN5, SPP1, **Table 2**; MMP3, TIMP1, THBS4, **Supplementary Table 2**) were significantly higher expressed in the lungs of non-healthy harbor porpoises.

**Table 2:** Top 20 of the significant DETs in the lungs of non-healthy harbor porpoises.

Gene ID	Gene name	FC_log2_	pval_FDR_	Function
Upregulated				
IGHG1 (LOC136143280)	Immunoglobulin Heavy Constant Gamma 1	3.79	1.16 × 10^-19^	immune response
JCHAIN	Joining Chain of Multimeric IgA and IgM	3.74	1.16 × 10^-19^	immune response
CXCL8	Interleukin 8	3.12	3.64 × 10^-15^	major mediator of the inflammatory response
IGKC (LOC136133810)	Immunoglobulin Kappa Constant	2.77	3.15 × 10^-10^	immune response
IGHV3-23 (LOC136143286)	Immunoglobulin Heavy Variable 3-23	2.67	1.35 × 10^-08^	immune response
LTF	Lactotransferrin	2.49	2.04 × 10^-07^	major iron-binding, multifunctional, exocrine fluid protein
SFTPA (LOC136136182)	Surfactant Protein A	2.45	5.45 × 10^-08^	essential for normal respiration by lowering surface tension
IGHV4-38-2 (LOC136143293)	Immunoglobulin Heavy Variable 4-38-2	2.38	7.72 × 10^-07^	immune response
SPP1	Secreted Phosphoprotein 1	2.30	2.37 × 10^-06^	major non-collagenous bone protein
SFTPB	Surfactant Protein B	2.25	2.75 × 10^-06^	essential for lung function and stability
MMP9	Matrix Metallopeptidase 9	2.05	1.48 × 10^-06^	breakdown of ECM and collagens
TNC	Tenascin C	2.02	1.96 × 10^-05^	extracellular matrix protein
TNFRSF17	TNF Receptor Superfamily Member 17	1.99	2.06 × 10^-04^	humoral immune system, activation of NF-kappa-B and JNK
CCN5	Cellular Communication Network Factor 5	1.90	1.28 × 10^-04^	bone turnover modulation
CXCL17	C-X-C Motif Chemokine Ligand 17	1.86	6.01 × 10^-04^	mucosal, potent antimicrobial chemokine
PIGR	Polymeric Immunoglobulin Receptor	1.83	0.001	microbial scavenger
GPNMB	Glycoprotein Nmb	1.77	3.66 × 10^-04^	transmembrane glycoprotein (type I)
SLC2A3 (LOC136130342)	Solute Carrier Family 2 Member 3	1.73	0.001	glucose transporter
SELP	Selectin P	1.68	3.41 × 10^-05^	immune-responsive cell adhesion molecule
SBSN	Suprabasin	1.68	0.006	located in extracellular exosome
Downregulated				
NREP	Neuronal Regeneration Related Protein	-1.66	0.001	neural function and regulation of alveolar generation
B2M (LOC136118455)	Beta-2-Microglobulin	-1.54	0.005	antigen presentation
NRXN1	Neurexin 1	-1.50	0.001	cell-cell-interaction, signal transmission
ADAMTS2	ADAM Metallopeptidase with Thrombospondin Type 1 Motif 2	-1.46	0.008	processes procollagens
KCP	Kielin Cysteine Rich BMP Regulator	-1.42	0.023	positive regulation of BMP signaling pathway
SPON1	Spondin 1	-1.38	0.001	major vascular smooth muscle cell factor, cell adhesion
MFAP2	Microfibril Associated Protein 2	-1.25	0.034	major antigen of elastin-associated microfibrils
COL3A1	Collagen Type III Alpha 1 Chain	-1.24	0.043	fibrillar collagen
BCHE	Butyrylcholinesterase	-1.17	0.048	detoxification of chemicals
TF (LOC136122826)	Transferrin	-1.06	0.043	iron transport

Given are the associated gene ID and gene name, logarithmic fold change (FC_log2_), the FDR-corrected p-value (pval_FDR_) and short description of the main function. If the gene IDs for the transcript were identified as a locus (LOC) in the transcriptomic analysis, they are given in the brackets. The associated gene ID was inferred via the databases of NCBI Gene and UniProt. For the short description of the main function, the databases of UniProt and GeneCards were used. Only 10 DETs were identified as significantly downregulated with the cut-offs pval_FDR_ ≤ 0.05, FC_log2_ ≤ -1 and mean TPM_non-healthy_ ≥ 5.

No overrepresented biological processes could be identified for the significantly downregulated DETs in the lung, as the number of DETs was too low (**Table 2**). Five downregulated DETs were involved in extracellular matrix processes (ADAMTS2, KCP, SPON1, MFAP2, COL3A1; **Table 2**).

### 3.2 qRT-PCR of dysregulated transcripts supports the lung transcriptome results

We performed a qRT-PCR on selected transcripts to confirm the transcriptome results in a larger sample size. Mean relative gene expression of JCHAIN, GPNMB and OLR1 was higher in non-healthy compared to the healthy harbor porpoises (**Supplementary Figure 2**). QPCT showed lower expression in non-healthy harbor porpoises (**Supplementary Figure 2**). As expected with wild, unmanaged animals, the relative gene expression of all transcripts showed some individual variation within the condition groups. However, the relative gene expression showed a similar regulation in non-healthy and healthy harbor porpoises as the gene expression calculated in the transcriptome analysis.

### 3.3 Increased stress responses and reduced tissue structure maintenance in the skeletal muscles

**Figure 2:**
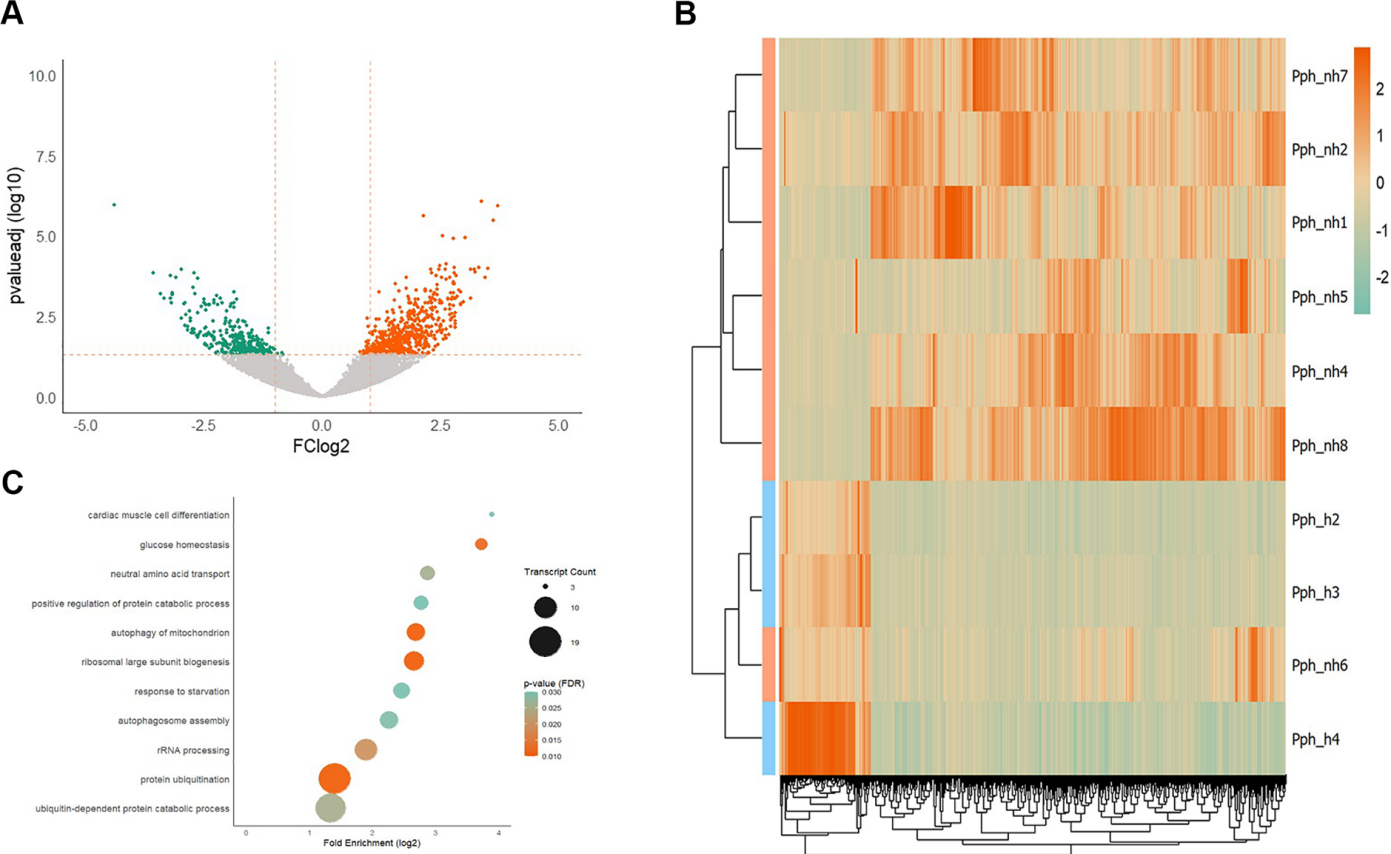
Differential analysis of the muscle. **(A)** Volcano plot of the significantly dysregulated transcripts in the muscle of non-healthy compared to healthy harbor porpoises. Significantly upregulated transcripts are highlighted in orange and downregulated transcripts in green. The x-axis shows the FC_log2_ and the y-axis depicts the FDR-corrected p-value (log10-transformed). The applied cutoffs are highlighted by dashed orange lines (FC_log2_ ≥ 1/≤ -1 and pval_FDR_ ≤ 0.05). Transcripts were considered significantly dysregulated when pval_FDR_ ≤ 0.05, FC_log2_ ≥ 1/≤ -1 and mean TPM_non-healthy_ ≥ 5. **(B)** Hierarchical clustering of the significant DETs in the muscle of non-healthy (apricot) and healthy (light blue) harbor porpoises. Upregulated transcripts are highlighted in orange and downregulated transcripts in green (see legend for FC_log2_ values). **(C)** Overrepresentation analysis of GO-Slim terms in “Biological Process” with PANTHER (reference: human) showed significant enrichment for upregulated transcripts, but not for downregulated transcripts. The y-axis shows the log2-transformed fold enrichment. Size of the dot represents the number of identified transcripts and the color of the dots indicates the significance level from high (orange) to low (green).

In the skeletal muscle, 17,540 transcripts were expressed in at least one animal. Of these, a total of 525 DETs were significantly higher expressed in in the skeletal muscle of non-healthy harbor porpoises, while 115 DETs showed a reduced expression compared to healthy individuals (**Figure 2A**). One bycaught, juvenile harbor porpoise, categorized as non-healthy (Pph_nh6), clustered with the healthy cohort, when comparing overall individual expression profiles of the significant DETs (**Figure 2B**).

Most enhanced pathways were associated with selective degradation processes (autophagy of mitochondrion, autophagosome assembly, protein ubiquitination, ubiquitin-dependent protein catabolic process; **Figure 2C**). Few regenerative processes, such as cardiac muscle cell differentiation, ribosomal large subunit biogenesis and rRNA processing, were elevated (**Figure 2C**). Also, muscles of non-healthy harbor porpoises displayed upregulation of processes pointing to nutrient stress, including glucose homeostasis and response to starvation. Likewise, several highly upregulated transcripts had a function in metabolism (AMPD3, IP6K3, PRODH, IRS2, PFKFB3, **Table 3**), with many involved in lipid metabolism (e.g., ADIPOR2, FASN, LPIN1, ACSL4, GPAT4, **Supplementary Table 3**). Additionally, a considerable number of DETs played a role in the nutrient stress-mediated response of the FoxO signaling pathway (hsa04068, **Supplementary Figure 3A**). These enhanced pathway responses included the cell cycle and DNA repair (CDKN1A, GADD45A, GADD45B, GADD45G), glycolysis (PCK2) and muscle atrophy (FBXO32) among others, while apoptosis was reduced (TNFSF10). In addition, several significantly upregulated transcripts in non-healthy harbor porpoises were involved in the HIF1 signaling pathway which modulates responses to oxygen deficit (IL6R, INSR, IGF1R, AKT2, MKNK2, EIF4EBP1, PRKCA, CAMK2B, HMOX1, HK2, PFKFB2, CDKN1A; hsa04066, **Supplementary Figure 3B**). Transcripts implicated in detoxification reactions via glutathione were also found with higher expression profiles in non-healthy harbor porpoise muscles (CHAC1, GPX3, GLUL, GSTT3, CTH, **Supplementary Table 3**).

**Table 3:** Top 20 of the significant DETs in the muscles of non-healthy harbor porpoises.

Gene ID	Gene name	FC_log2_	pval_FDR_	Function
Upregulated				
AMPD3	Adenosine Monophosphate Deaminase 3	3.71	1.22 × 10^-06^	branch point in the adenylate catabolic pathway, muscular isoform
CDKN1A	Cyclin Dependent Kinase Inhibitor 1A	3.62	3.45 × 10^-06^	mediator of stress-responsive cell cycle G1 phase arrest
GADD45G	Growth Arrest and DNA Damage Inducible Gamma	3.50	1.03 × 10^-04^	environmental stress response
TRIM63	Tripartite Motif Containing 63	3.44	2.00 × 10^-04^	E3 ubiquitin ligase, muscle atrophy
KLHL30	Kelch Like Family Member 30	3.37	8.38 × 10^-07^	proteasome-mediated ubiquitin-dependent process
KCNIP2	Potassium Voltage-Gated Channel Interacting Protein 2	3.36	1.47 × 10^-25^	calcium-binding modulator of channel density and inactivation
H1-2	H1.2 Linker Histone, Cluster Member	3.24	1.38 × 10^-04^	nucleosome structure and organization of chromatin
TNFRSF12A	TNF Receptor Superfamily Member 12A	3.21	1.13 × 10^-04^	extrinsic apoptotic signaling pathway and wound healing
ANKRD33B	Ankyrin Repeat Domain 33B	3.15	1.10 × 10^-04^	unknown, associated with muscle cells
IGHG4 (LOC136143276)	Immunoglobulin Heavy Constant Gamma 4	3.14	0.001	antibacterial humoral response and complement activation
IL6R	Interleukin 6 Receptor	3.02	1.13 × 10^-05^	immune response, cell growth and differentiation regulation
CABYR	Calcium Binding Tyrosine Phosphorylation Regulated	2.94	0.001	calcium-binding
MAFF	MAF BZIP Transcription Factor F	2.92	0.001	dimeric transcriptional repressor, cellular stress response
IP6K3	Inositol Hexakisphosphate Kinase 3	2.92	0.001	converts inositol hexakisphosphate to diphosphoinositol pentakisphosphate
PRODH	Proline Dehydrogenase 1	2.88	4.86 × 10^-04^	initial step in proline degradation
SDC4	Syndecan 4	2.85	0.001	intracellular signaling receptor involved in exosome biogenesis
IRS2	Insulin Receptor Substrate 2	2.83	1.76 × 10^-04^	multifunctional signal transduction adapter
S100A12	S100 Calcium Binding Protein A12	2.82	0.006	inflammation and immune response
LYPD3	LY6/PLAUR Domain Containing 3	2.81	0.003	laminin binding activity
PFKFB3	6-Phosphofructo-2-Kinase/Fructose-2,6-Biphosphatase 3	2.81	0.001	glycolysis regulation
Downregulated				
COL1A2	Collagen Type I Alpha 2 Chain	-2.91	0.001	fibril-forming collagen
COL3A1	Collagen Type III Alpha 1 Chain	-2.73	3.97 × 10^-04^	fibril-forming collagen
ITM2A	Integral Membrane Protein 2A	-2.69	0.001	osteo- and chondrogenic differentiation
CAPN6	Calpain 6	-2.50	0.001	calcium-dependent cysteine protease responsive to oxidative stress
COL1A1	Collagen Type I Alpha 1 Chain	-2.41	0.01	fibril-forming collagen
G0S2	G0/G1 Switch 2	-2.39	0.001	extrinsic apoptotic signaling pathway
FN1	Fibronectin 1	-2.30	0.004	cell adhesion and migration, soluble dimeric and anchored multimeric form
TMEFF2	Transmembrane Protein with EGF Like and Two Follistatin Like Domains 2	-2.28	0.001	transmembrane protein
MEST	Mesoderm Specific Transcript	-2.20	0.047	development
OLFML1	Olfactomedin Like 1	-2.15	0.001	signal transduction
NID2	Nidogen 2	-2.12	0.001	collagen-binding, basement membrane structure maintenance
MARCKS	Myristoylated Alanine Rich Protein Kinase C Substrate	-2.03	0.02	motility- and phagocytosis-modulating proteinkinase C substrate
CALML4	Calmodulin Like 4	-2.03	0.002	myosin head/neck binding
RXRG	Retinoid X Receptor Gamma	-2.03	0.009	antiproliferative retinoic acid receptor
LXN	Latexin	-2.02	0.011	zinc-dependent metallocarboxypeptidase protein inhibitor
FAP	Fibroblast Activation Protein Alpha	-2.01	0.024	extracellular matrix degradation, wound healing and inflammation
ITGA6	Integrin Subunit Alpha 6	-1.96	0.005	cell surface adhesion and signaling
F2R	Coagulation Factor II Thrombin Receptor	-1.95	0.013	regulation of thrombotic response and of proinflammatory cytokines
CD38	CD38 Molecule	-1.94	0.024	messenger for intracellular calcium activation and glucose-induced insulin secretion
TSPAN12	Tetraspanin 12	-1.92	0.001	cell surface receptor, regulator of membrane proteinases

Shown are the gene ID and gene name, FC_log2_, pval_FDR_ and a short description of the main function (cut-offs for consideration: pval_FDR_ ≤ 0.05, FC_log2_ ≤ -1 and mean TPM_non-healthy_ ≥ 5). Transcripts identified with a locus ID (LOC) are given in the brackets and the associated gene ID inferred from the NCBI Gene and UniProt databases. UniProt and GeneCards were used to define the main function.

Multiple DETs within a single gene family exhibited concordant expression, suggesting coordinated family level regulation (**Supplementary Table 3**). This included the Kelch like gene family (6 DETs), solute carrier family (16 DETs), transmembrane-associated TMEMs (7 DETs) and of two transcription factor gene families (3 Maf transcription factors and 9 zinc finger transcription factors).

For the muscle, no significantly decreased pathways were identified with the GO and KEGG analysis. Collagen transcripts (COL1A2, COL3A1, COL1A1, **Table 3**), cell structure- and adhesion-associated DETs (e.g., CAPN6, FN1, NID2, MARCKS, FAP, ITGA2, CD38, TSPAN12; **Table 3**) were among the most significantly decreased transcripts in relation to healthy animals.

### 3.4 Seven transcripts show similar high dysregulation in the lung and muscle

**Figure 3:**
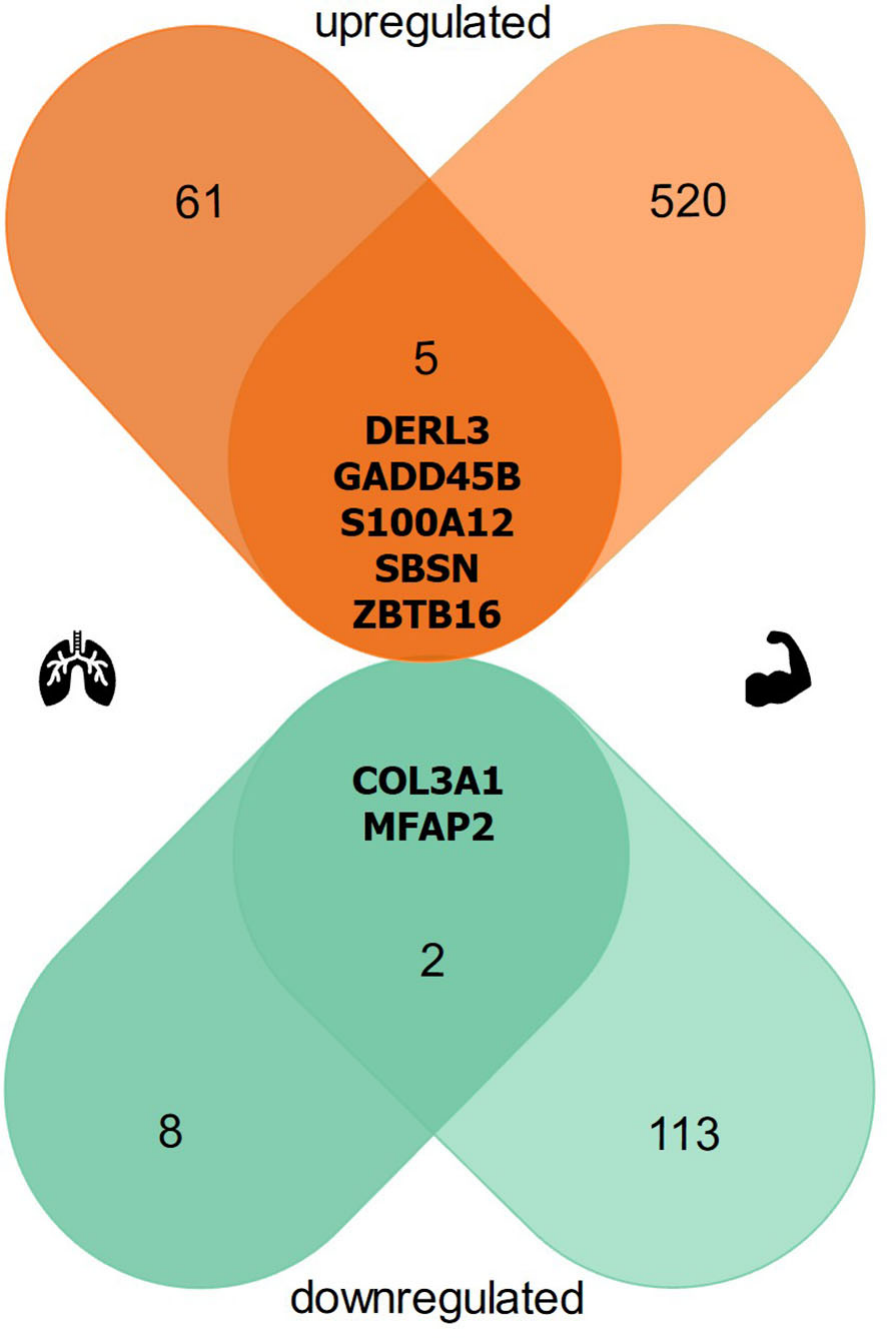
Venn diagram of simultaneously dysregulated transcripts in the lung and muscle of non-healthy harbor porpoises. In non-healthy harbor porpoises, five transcripts were significantly upregulated in the lung and muscle (DERL3, GADD45G, S100A12, SBSN, ZBTB16, depicted in orange), while two transcripts showed significant downregulation overlapping in both tissues (COL3A1, MFAP2, highlighted in green). 61 upregulated transcripts and eight downregulated transcripts were found only in the lung and 520 upregulated transcripts and 113 downregulated transcripts were identified only in the muscle.

Comparing the significantly differentially expressed transcripts of the lungs to those of the muscles in non-healthy harbor porpoises with healthy harbor porpoises, we identified an overlap of seven dysregulated transcripts (**Figure 3**). Five DETS were found upregulated and two downregulated in both tissues, with a total of four DETs also found among the 20 most dysregulated transcripts (up: SBSN, S100A12; down: COL3A1, MFAP2; **Tables 2**, **3**). Further concordant DETs were found in proteasome-associated DERL3 and stress-mitigating GADD45B and ZBTB16.

### 3.5 *De novo* assembly shows dysregulation of several uncharacterized ncRNAs

To identify species-specific, potentially novel transcripts, we back-mapped the lung transcriptomes as well as the corresponding muscle transcriptomes to the *de novo* transcriptome assembly. The *de novo* assembly was generated using transcriptomes of four tissues (lung, muscle, brain, liver). A total of 204,013 Trinity transcripts were assembled, of which 148,936 were annotated with the Trinotate pipeline, using Blast and Diamond. Statistics of the *de novo* assembly are given in **Table 4**. We employed strict cutoffs for the differential expression analysis (pval_FDR_ ≤ 0.05, FC_log2_ ≥ 1 or ≤ -1, mean TPM_non-healthy_ ≥ 5) and only considered Trinity transcripts that showed a TPM ≥ 0 in at least half of the individuals in each condition group (healthy, non-healthy). This resulted in 24 significantly upregulated and 9 downregulated Trinity transcripts in the lung (**Supplementary Table 4**). In the muscle, 390 upregulated and 50 downregulated Trinity transcripts were identified (**Supplementary Table 5**). We identified several potentially novel transcript variants, lncRNAs and ncRNAs in the lung and muscle (**Supplementary Tables 4**, **5**). In the lung, one downregulated Trinity transcript in non-healthy harbor porpoises was identified as ncRNA (LOC112408907), while in the muscle, a total of nine Trinity transcripts were identified as ncRNAs or lncRNAs (LOC116758475, LOC114484449, LOC137228934, LOC137231800, LOC109549532, LOC109549603, LOC117309420, LOC141276952, LOC116665024; **Supplementary Table 5**). Two upregulated Trinity transcripts of the muscle were determined to be an uncharacterized cetacean mRNA or protein (LOC118894103, LOC138842886; **Supplementary Table 5**). Lastly, the differential *de novo* assembly analysis in the muscle revealed four upregulated and seven downregulated Trinity transcripts that could not be further identified by Blast, Diamond or NCBI Blastn/Blastp with the applied cut-offs (**Supplementary Table 5**).

**Table 4:** Statistics of the *de novo* assembly.

Trinity statistics		
Total trinity transcripts	204,013	
Total trinity genes	129,859	
GC content	47.87	
	All transcript contigs	Longest isoform per gene
Total assembled bases	359,234,987	167,863,701
Mean contig length	1,760.84	1,292.66
Median contig length	1,057	802
N50	2,719	1,602
BUSCO		
	Cetartiodactyla	Mammalia
Complete	76.50%	78.00%
Duplicated	53.10%	55.10%
Fragmented	4.40%	5.60%
Missing	19.10%	16.40%

BUSCO, Benchmarking Universal Single-Copy Orthologs (highly conserved gene orthologs, expected to be expressed in all Cetartiodactyla/Mammalia). For the *de novo* assembly, the Trinity and Trinotate pipeline was used. General statistics of the assembly are shown (total trinity transcripts, total trinity genes, GC content, annotated transcripts) and the pre-assembly quality was analyzed with the statistics of the mean contig length, median contig length and N50 for all transcripts and the longest isoform per gene. Post-assembly quality was inferred from identified orthologs in Cetartiodactyla and Mammalia using BUSCO.

## 4 Discussion

Harbor porpoise populations inhabiting the North and Baltic Seas are experiencing increasing impacts due to a variety of human activities, including underwater-radiated noise and chemical pollution. This can have adverse effects on immunocompetence, resulting in an increased susceptibility to pathogens and infectious diseases. Harbor porpoises suffer from parasitic and bacterial infections of the respiratory tract and associated lesions which may impair the lung function. Since harbor porpoises develop respiratory lesions to a severity which is not found in humans and terrestrial animals, it is not known how they deal with effects on reduced oxygen uptake as diving mammals. As an air-breathing, fully aquatic mammal and apex predator, this may have a negative effect on their viability, if receiving organs, such as the skeletal muscles, are supplied with insufficient oxygen, as they consume enormous amounts of oxygen and energy to enable diving ability and foraging.

### 4.1 Broad activation of immune and surfactant processes and ECM-modulating transcripts indicate effort to mitigate pathogenic insults

Harbor porpoises suffering from severe nematode infestations and associated bacterial bronchopneumonia exhibited an expected, pronounced immune response in the lung tissue. As one of the first interfaces of the organism with its environment, the respiratory tract functions as a primary responding tissue to detrimental viral, bacterial and pathogenic insults, thus employs a wide array of innate and adaptive immune responses (49). The high expression of immunoglobulins and leukocytes suggests increased opsonization of pathogens (50), while inflammatory cytokines, such as CXCL8 and CXCL17, indicate an acute activation of adaptive immune responses in the lungs of non-healthy harbor porpoises (51).

Large nematodes within the tissue not only disrupt tissue integrity, but also cause inflammation and can facilitate bacterial or viral infection. In mammals, this typically induces a T helper 2 cell (Th2) immune response to manage nematode load and tissue regeneration (52). While we observed some upregulation of genes in the lungs of non-healthy harbor porpoises that are also implicated in the Th2 immune response, such as IGHG1, MMP3, MMP9 and TIMP1 (52, 53), our results do not suggest a definite activation of the Th2 response. This is consistent with a recent study which observed a Th2 immune response in harbor porpoises suffering from mild to moderate lungworm burden, but not in severe cases (54). Since our distinction between non-healthy and healthy porpoises was solely based on severity of nematode burden in the respiratory tract, our non-healthy animals likely represent severe disease cases. Whether this is caused by overwhelming and prolonged nematode burden or exacerbating immune challenge and subsequent compromise remains to be elucidated.

To enable influx of recruited immunocytes and other cell types necessary for wound healing to the inflamed or infested area, matrix metallopeptidases readily degrade the extracellular matrix and its components (55). This turnover of the ECM can be indicated by the ratio of MMPs to TIMP1, their inhibitor, with increased MMPs resulting in higher degradation rates. Here, non-healthy harbor porpoises appear to show higher ECM degradation, as indicated by higher upregulation of MMP3 and MMP9 compared to TIMP1 and dysregulation of ECM processes. Although tissue degradation is also an important step to enable tissue remodeling and regeneration, non-healthy harbor porpoises showed a marked decrease in expression of type III collagen, a main constituent of healthy lungs (56). It is therefore plausible that harbor porpoises with exceeding nematode burden and poor pulmonary health may struggle to adequately and swiftly replace damaged or degraded tissue which can lead to accelerated permanent tissue loss and functional impairment.

Within the respiratory system, the surfactant mucus lining facilitates respiration, but also represents an additional layer of immune protection (57). Likewise, lungs of non-healthy harbor porpoises displayed higher expression of surfactant transcripts with a primary role in host defense and airway clearance, and respiration and hypertension relief (57, 58). Surfactant is composed of ~85% phospholipids and has a high turnover rate in healthy lung tissue that can be accelerated or inhibited in compromised lungs (59). However, disrupted surfactant production can have negative consequences if recycling capacity is exceeded and can manifest itself in the form of atherosclerosis-like symptoms (60, 61). Disease-associated surfactant alterations in lipid composition have also been found to affect lung structure and mechanical function in dolphins (62). In line with this, lungs of non-healthy harbor porpoises exhibited upregulation of several transcripts involved in lipid-associated atherosclerosis, hinting at pathologically altered regulation.

Often considered as a passive diffusion organ, the lungs are metabolically active (63). Despite being one of the most well-oxygenated tissues, the lungs largely utilize lactate as energy substrate for aerobic glycolysis, potentially to reduce oxygen consumption and preferably distribute oxygen to distant organs (64). Here, no transcripts were significantly dysregulated that indicated higher lactate conversion. However, the observed elevation of carbohydrate transport suggests an increased cellular glucose metabolism, as host defense, surfactant production and respiration are energy-intensive processes (63, 64). This is in line with previous studies in other mammals which showed that the lung glucose consumption in part exceeds that of other highly metabolic organs (63, 64).

### 4.2 Enhancement of HIF1 and stress-responsive signaling, immune activation and cell cycle checkpoints point to downstream effect of lung lesions in the skeletal muscles

The comparative muscle transcriptome analysis indicates an effect from the oxygen-uptaking lung to the oxygen-demanding muscle of harbor porpoises with compromised respiratory health. Muscles exhibited enhanced stress signaling pathways and transcripts that pointed to elevated endurance of cumulative stressors, including hypoxia, starvation, chemical stress and inflammation, respectively. Enrichment of the key hypoxia-mediating HIF1 signaling pathway as well as of the stress-responsive FoxO signaling pathway implicates that these possibly are important signaling pathways involved in the muscle response of harbor porpoises with a compromised lung health. Here, HIF1-regulated transcripts were significantly elevated in non-healthy porpoises that shift energy production to glycolysis, a hallmark feature of hypoxic and inflammatory states and which is utilized by cetacean muscles to fuel diving under depleting oxygen availability (65–67). The muscle is highly metabolic, serves as nutrient and oxygen storage in whales, but also consumes enormous amounts of energy to fuel energetically-costly diving and an aquatic lifestyle (68, 69). Additionally, harbor porpoises are known to have a high metabolic field rate, resulting in constant foraging (30), but do not possess large energy reserves due to their relatively small size and muscle mass, putting them at higher risk of starvation (70).

Indicating similarities to studies of patients with lung diseases such as COPD (71–73), the muscles of non-healthy harbor porpoises displayed enrichment of autophagy and muscle atrophy pathways. We also observed reduced cell integrity which can be exacerbated by both pulmonary disease and insufficient oxygen (71, 73, 74). While autophagy is a normal process in the healthy musculature that enables regeneration and muscle growth, under metabolic deprivation or stress, disproportionate regulation leads to utilization of muscle protein to fuel essential functions (75, 76). This may further aggravate this incompetence in harbor porpoises (30, 34) and moreover, disables harbor porpoises to avoid other stressors, such as underwater-radiated noise which causes spontaneous deeper diving or rapid swim speed acceleration (35, 36).

In accordance with our previously published results (77), protein synthesis and regeneration-associated transcripts were elevated, while muscles seemingly also employed mitotic cell cycle checkpoints. This coordinated response may aid in minimizing DNA damage and reduces accumulation of defective cells which is important since muscles are a post-mitotic tissue that needs to adapt quickly to environmental changes (75, 78, 79).

While the muscle of non-healthy harbor porpoises showed elevated anaerobic metabolism and higher atrophic states, the transcriptomic analysis hinted at a decreased leak respiration and more efficient energy utilization by lower expression of uncoupling protein UCP3 (FC_log2_ = -2.04; pval_FDR_ = 0.069) and elevation of its regulator NPY (FC_log2_ = 2.04; pval_FDR_ = 0.035). UCP3 has not yet been detected in cetaceans. Studies have described its pseudogenized paralog UCP1 in cetaceans and postulated a function in lipid processes (80, 81), however the integrity and function of cetacean UCP3 remains to be elucidated.

### 4.3 Cross-tissue dysregulated transcripts are affected by environmental stressors

Our systemic transcriptome analysis revealed five upregulated and two downregulated DETs in the lungs and muscles that were simultaneously dysregulated in non-healthy harbor porpoises. The concordant regulation of this DET subset in both tissues highlights their potential role in the extrapulmonary, pathogenic processes extending beyond the lungs. This included upregulated SBSN which has been associated with hypoxic environments (82). SBSN was also found elevated after endurance exercise in sled dog muscles (83), suggesting a causal link between prolonged muscle exercise capacity and inadequate oxygen concentration. Further supporting a connected systemic response to an impaired lung function, we observed a significant upregulation of stress signaling transcripts GADD45G and ZTBT16. While GADD45G is swiftly upregulated upon genotoxic and environmental stress (84), ZBTB16 is a transcription factor in response to cold stress and modulates higher utilization of fatty acid metabolism and glycolytic capacity (85). Elevated serum levels of S100A12 are indicative of the inflammatory progression of acute and chronic lung diseases (86), while in skeletal muscle it is not predominantly found or expressed. Hence, the upregulation in the lung and muscle of non-healthy harbor porpoises may stem from the compromised respiratory system and contribute to multi-organ dysfunction in the muscle. The systemic dysregulation of structural transcripts (COL3A1, MFAP2) and of transcripts associated with the ubiquitin-dependent proteasome (DERL3) points to limited tissue remodeling and ECM structure (87, 88), which is consistent with observations in other studies of extrapulmonary morbidities of respiratory diseases (72, 73, 89).

However, the lungs and the skeletal muscles are highly vascularized tissues. Thus, the question remains as to what extent the simultaneous dysregulation of these DETs is attributable to the blood circulation from other organs, since non-healthy harbor porpoises suffered from several diseases, including lesions in the pulmonary blood vessels. Although we cannot be certain that the observed regulation is exclusively driven by the pathological lung lesions, the concordant dysregulation of several transcripts highlights how disease states induce a coordinated, systemic injury response in harbor porpoises.

### 4.4 *De novo* assembly reveals several potentially regulatory ncRNAs in the lung and muscle

The *de novo* assembly of the harbor porpoise lung and the muscle displayed overall similarly dysregulated biological processes in non-healthy harbor porpoises like the differential analysis (data not shown). However, several significantly dysregulated Trinity transcripts encoded for non-coding (nc) RNAs, which may possess an important role in rapid immunoregulation and time-sensitive responses to environmental or DNA-damaging stimuli. Similar observations have been stated in studies of humans and mice, which found ncRNAs to have distinct spatiotemporal expression profiles that can rapidly change upon external insults and disease to alter immune cell composition and responses (90, 91). Therefore, the function and effect of the dysregulated ncRNAs in the lungs and muscles of non-healthy harbor porpoises should be subject of future *in vitro* studies, since they could have a profound immunoregulatory effect in response to the observed lung lesions and systemic reaction from the muscles. Two significantly upregulated Trinity transcripts in the lung of non-healthy harbor porpoises originated from pathogenic helminths (data not shown), emphasizing the severity of parasite accumulation in non-healthy harbor porpoises (92). Furthermore, several Trinity transcripts that were significantly upregulated in the muscles of non-healthy harbor porpoises did not have a definite or unique match with other orthologs when analyzed with Blast, Diamond and NCBI Blast (as per our definition and cut-offs), thus potentially describe novel transcript variants. However, methods used for *de novo* assemblies and RNA-Sequencing can lead to an overestimate of identified transcripts. While we employed strict cut-offs and applied necessary precautionary filtering of the results to reduce such inconvenient effects, it is still possible that some of these variants may stem from overestimation or transcriptional noise amplified by the assembly. Another restricting factor is that reference databases for non-model organisms, such as marine mammals, are often incomplete and with substantial blanks which is due to the challenges of access to the animal, sampling methods and experimental limitations. Therefore, the definite classification of these putative transcripts and their function have to be studied in greater detail and be verified in further molecular studies.

### 4.5 Limitations

The samples used in this study are from necropsies of wild harbor porpoises that were dead for under 24 hours. Lungs act as vital interface between the animal and its environment, thus are constantly exposed to external impacts which might result in a more rapid degradation of the lungs compared to other tissues (e.g., muscle). Also, the observed pathological lesions in the respiratory tract and lungs may further accelerate tissue degeneration. In addition to decomposition state, access to marine mammal samples is generally very limited due to their aquatic, elusive lifestyle and conservation status (93). Within our two cohorts, there is an age class difference that overlaps with health status. This is in part also caused by high percentage of young harbor porpoises among post-mortem examined individuals and that adult harbor porpoises are rarely in good pulmonary health (20, 21). Harbor porpoises accumulate parasitic and pathogenic load with increasing food intake and advancing age (10, 22). Therefore, it cannot be entirely ruled out that age class may affect gene expression. The observed interindividual variation may also stem from differences in age, disease, and not known impacts prior to death which might result in diverse molecular signatures. Here, one juvenile individual classified as non-healthy (Pph_nh6, approx. one year old) groups with the healthy cohort in the comparative analysis of the muscle. Calves develop their musculature rapidly within the first year (94), while suffering from comparably less nematode presence than adults. This may result in downstream impacts that are not yet as severe as those in affected adults.

## 5 Conclusion

Harbor porpoises of the North and Baltic Seas increasingly suffer from pathological lesions in the lungs, that are caused and aggravated by accumulating parasitic load and associated secondary infections. These pathological lesions may disrupt lung tissue function, reducing oxygen uptake and supply to distant, receiving organs. This study provides deeper insights into the underlying compensatory and pathophysiological mechanisms of the lungs and locomotor muscles of harbor porpoises with a compromised pulmonary health. Our transcriptome analysis showed similar immune responses and defense mechanisms compared to humans suffering from lung diseases, suggesting that these reactions are conserved in mammals regardless of their distinct environments. Although nematode infections typically trigger a Th2 immune response, the lung transcriptomes did not show such expression profiles, which is consistent with previous observations of a reduced Th2 activation in harbor porpoise lungs with severe nematode infestation. Lungs of non-healthy harbor porpoises displayed higher expression of surfactant transcripts that mainly aid with respiration, and enhanced carbohydrate transport which may help to replenish diminishing energy reserves. Our results suggest a detectable systemic effect of the compromised lung on the locomotor muscles, resulting in a response to multiple impacts, such as inflammation, hypoxia and starvation. Such effects on the muscles have also been observed on humans suffering from lung diseases. A swift, but tightly regulated switch between regeneration and degradation in the muscles may represent an attempt to alleviate excessive muscle atrophy to uphold tissue function. However, mitigating cumulative impacts is strenuous and requires substantial amounts of energy which may be challenging for non-healthy harbor porpoises. The *de novo* assembly revealed several highly dysregulated non-coding RNAs in the lungs and muscles of non-healthy harbor porpoises. Non-coding RNAs possess important regulatory functions, thus may cause fast spatiotemporal responses to distinct stimuli. Although some molecular immune responses in cetaceans are not well understood, this study provides relevant insights into cetacean immune responses to diseases and pathogens and their interaction with anthropogenic stressors, thereby supporting conservation efforts.

## Data Availability

The datasets presented in this study can be found in online repositories. The names of the repository/repositories and accession number(s) can be found below: https://www.ncbi.nlm.nih.gov/, PRJNA977857.
