# Supplementary figures and images for: Transcriptomic insights into the immune responses of the lung and muscle of non-healthy harbor porpoises (Phocoena phocoena)
Source: Front Immunol. 2026 Mar 9;17:1738836. doi: 10.3389/fimmu.2026.1738836 (PMC13006228; doi:10.3389/fimmu.2026.1738836)

## Slide 1
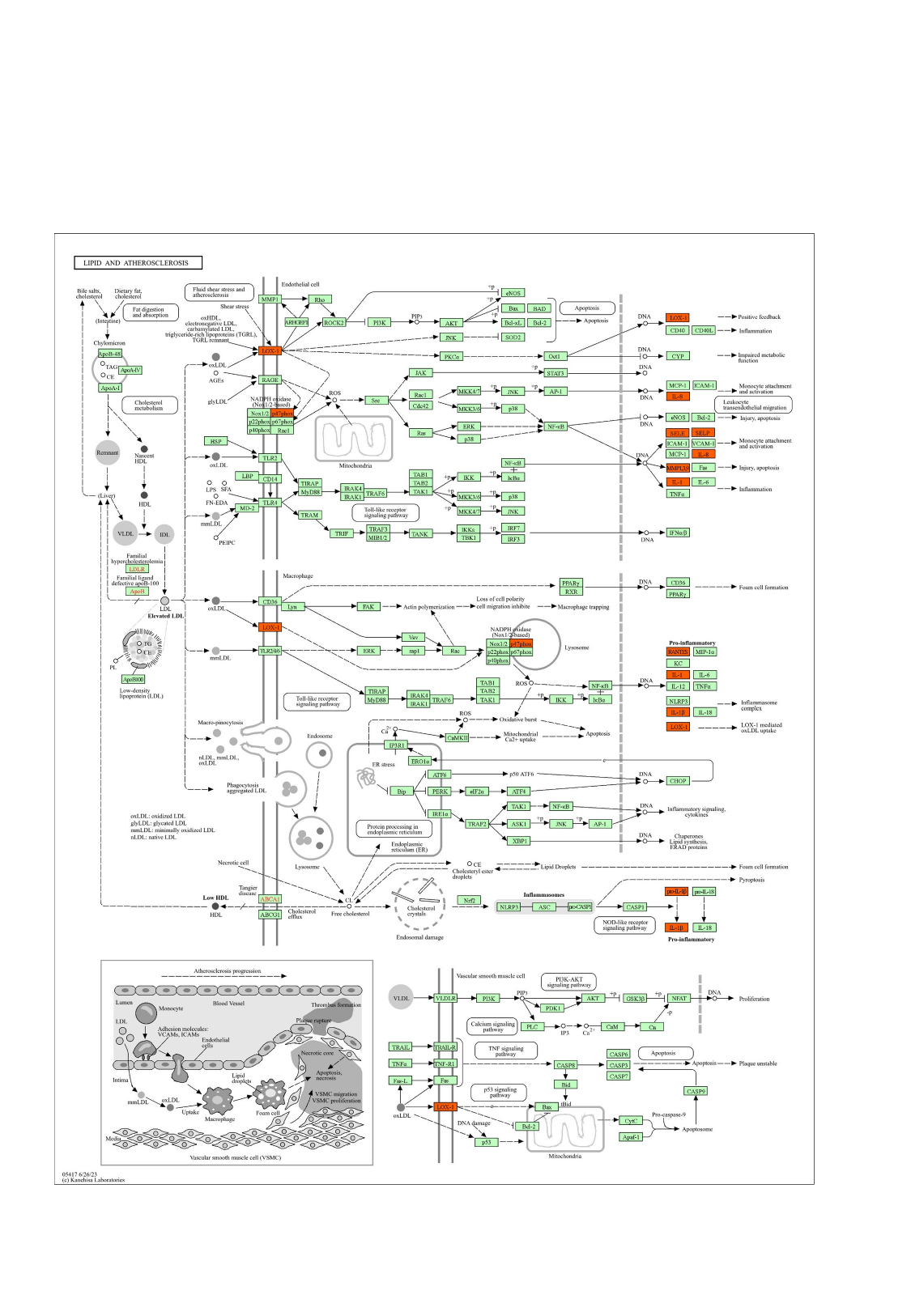

## Slide 2
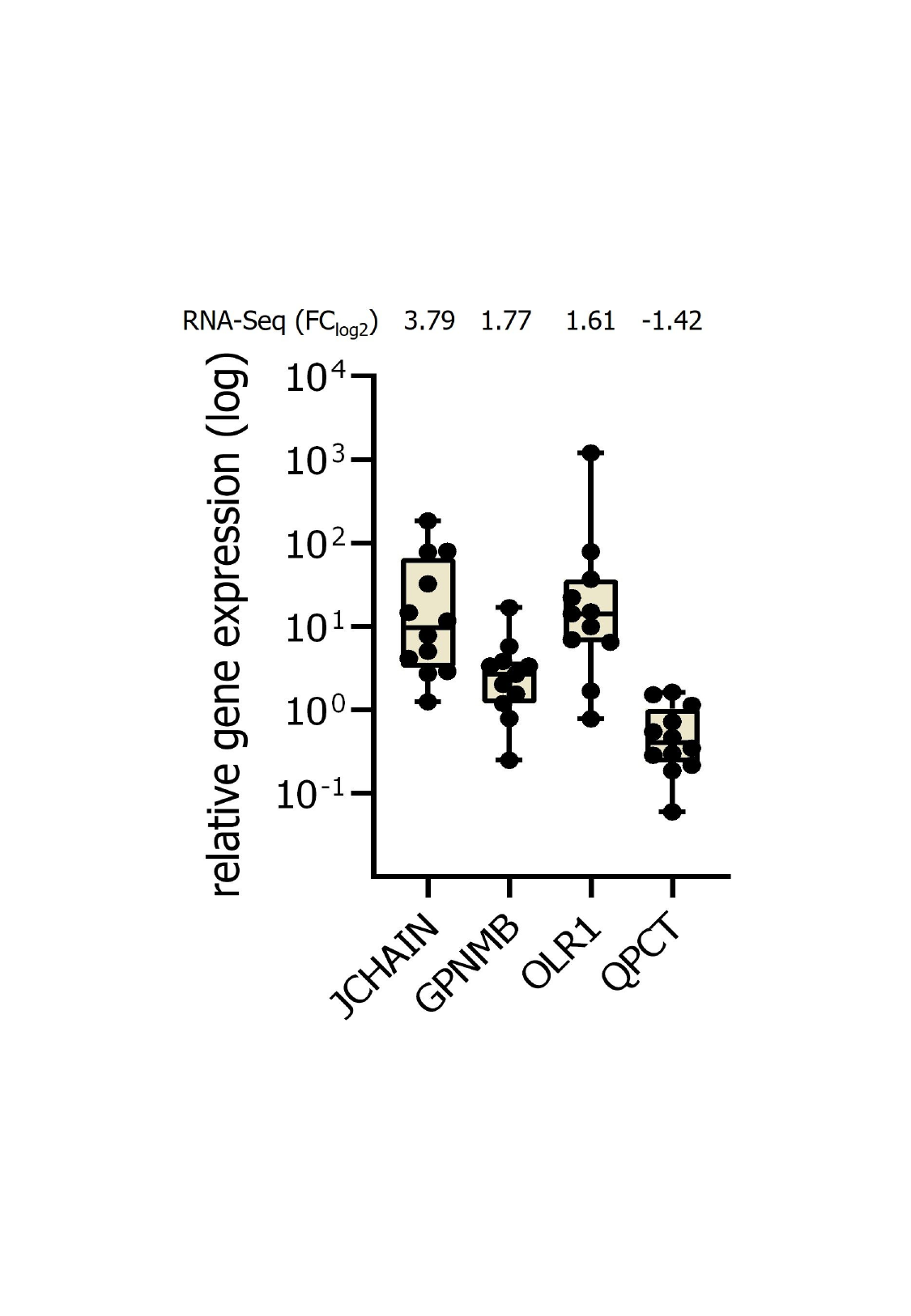

## Slide 3
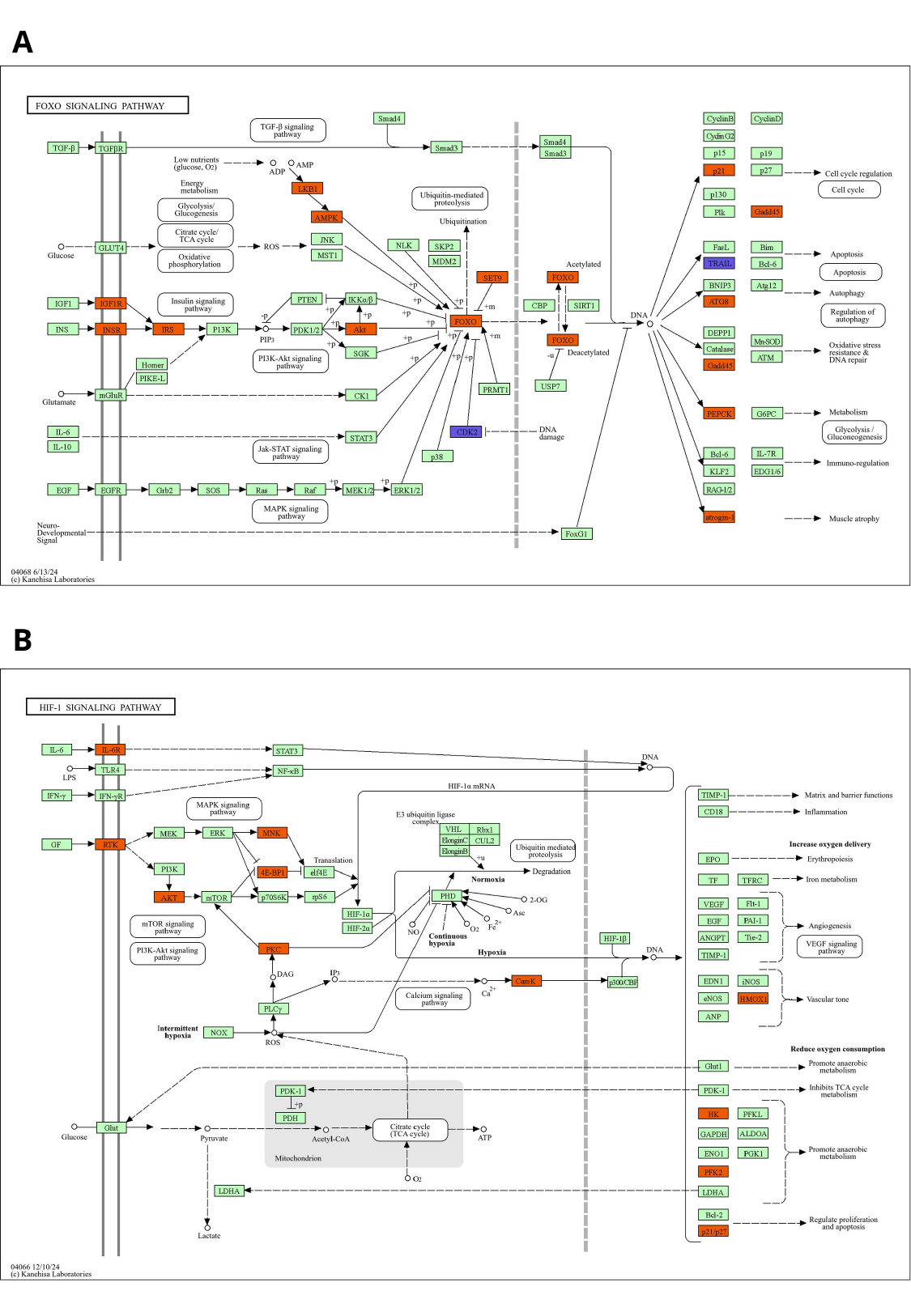

A
B

Supplement: Supplementary file 1 [file DataSheet1.zip › Supplementary_Figures_Transcriptomic insights into the immune responses of the lung and muscle of non-healthy harbor porpoises (Phocoena phocoena).pptx]
